# Utilizing Smart Televisions as Assistive Technology to Enhance Communication and Social Lives of Older Adults: Systematic Review

**DOI:** 10.2196/73050

**Published:** 2025-09-10

**Authors:** Jayde Langdon, Cristina Tugahan Cabansag, Alexis Grigoris, Way Kiat Bong

**Affiliations:** 1 Faculty of Health Sciences Western University London, ON Canada; 2 Department of Computer Science Faculty of Technology, Art and Design OsloMet – Oslo Metropolitan University Oslo Norway

**Keywords:** smart television, assistive technology, older adults, communications, social lives, elderly people, social contact

## Abstract

**Background:**

Over the past decade, the proportion of the world’s population aged ≥65 years has grown exponentially, presenting significant challenges, such as social isolation and loneliness among this population. Assistive technologies have shown potential in enhancing the quality of life for older adults by improving their physical, cognitive, and communication abilities. Research has shown that smart televisions are user-friendly and commonly used among older adults. However, smart televisions have been underutilized as assistive technologies.

**Objective:**

This study aimed to explore the state of the art in using smart televisions as assistive technologies to enhance communication and social interactions among older adults.

**Methods:**

The search was conducted following the guidelines for performing a systematic literature review, which included 6 databases, that are, the IEEE, ACM, Google Scholar, ScienceDirect, Engineering Village, and Springer. A range of keywords were used in different combinations, including “smart TV,” “older adults,” “elderly,” “communication,” “messaging,” “video call,” and “application.” A set of inclusion and exclusion criteria was defined before the search, and the screening was performed by 3 researchers. We analyzed the selected articles in accordance with the review’s aim and the established inclusion and exclusion criteria. None of the articles were subjected to quantitative synthesis because of the significant variations in the data measured.

**Results:**

After screening 2671 records from the abstract level to full text, 30 articles were identified as relevant studies, demonstrating both direct and indirect impacts on the social lives of older adults through the use of smart televisions as assistive technology. Some articles were part of the same or larger studies, which makes the number of actual projects even smaller. This indicates that smart televisions have been underutilized as assistive technologies for enhancing older adults’ communication and social lives. More than half of the articles proposed their own prototype, and these prototypes were primarily targeted for use at home, while some were targeted for use at geriatric care units or nursing homes. User involvement among older adults was high among the included articles, and some also included other users, such as health care personnel, administrative staff, and engineers. The included studies were mostly from Europe.

**Conclusions:**

This review highlights the potential of smart televisions as assistive technologies to enhance social connectivity among older adults, and identifies several research gaps. Most studies focus on short-term usability and are geographically limited to Europe. Future research should include longitudinal studies, explore diverse cultural attitudes, and focus on adaptive solutions for various health conditions. We hope this review will inspire research on smart televisions as assistive technologies, enhancing social interactions and quality of life for older adults.

## Introduction

### Background

Over the past decade, the proportion of the world’s population aged ≥65 years has grown exponentially. Researchers have taken an interest in this growth and have recognized the importance of exploring ways to accommodate the older adult community. The older adult population faces numerous challenges, ranging from physical decline to cognitive dysfunction. However, one of the most notable barriers that older adults face is social isolation [1]: the disconnection from friends, family, and the world around them. Loneliness is 1 of the 3 main factors leading to depression, a prominent condition among older people [2]. Social isolation and loneliness in old age have significant negative impacts on both physical and mental well-being [1]. As the world’s population of older adults grows, digital technology has increasingly and significantly influenced their daily lives. As we continue to age globally, it is critical that assistive technology is used to our advantage as a tool for ensuring that older adults age with a good quality of life. The World Health Organization defines assistive technology as an umbrella term for assistive products and their related systems and services [3]. Assistive technologies can help improve an individual’s functioning regarding cognition, communication, hearing, mobility, self-care, and vision; thus, enabling their health, well-being, inclusion, and participation. Although a large population of older adults hesitate to use technology, many of them, as well as their caregivers, benefit greatly from assistive technologies [3]. In many countries, research has shown that the number of users of assistive technologies seems to increase with age. A study in a subsample of a population aged 79 years showed that 64% of them had one or more assistive technologies, specifically for bathing [4]. Wheelchairs and stairlifts also enhance the mobility of older adults [5]. Hearing aids and automated lighting systems support older adults with both activities of daily living and independent activities of daily living [6]. At the same time, the rapid growth of the older adult population has also raised awareness of the importance of aging at home. Extending the time before older adults need to move into long-term care facilities or nursing homes can be achieved by creating a comfortable environment for aging at home. However, one of the challenges older adults face as they age is maintaining connections with friends, family, and society as a whole. Older adults who live at home are more susceptible to feelings of loneliness and depression [7]. Despite the development of information and communication technologies over the past decade, Choi and Lee [8] in their systematic literature review, found only 23 relevant studies that used information and communication technology to improve social participation among older adults and reduce their loneliness. On top of that, future research needs to look more into emerging technologies such as the Internet of Things, augmented reality, and virtual reality, as older adults are often not sufficiently addressed in the design and implementation of these technologies, as reported by Thangavel et al [9] in their scoping review. A study conducted in China on implementing smart homes to build connectivity discussed how most research and development of smart homes has actually focused on younger people, leaving little attention to older adults [10]. In addition, around 63% of older adults in Canada are either nonusers or basic users of the internet and digital technologies [11]. On average, 22.3% of the older population in Europe uses computers, and 16.8% are internet users, with large differences between age groups [12]. These disparities and usability barriers pose significant challenges in implementing assistive technology intended to enhance social interactions among older adults.

Tapia et al [13] argued that the introduction of computer-based technologies for social interactions has negatively affected the ways older adults interact with their family members. This can be attributed to a lack of technological knowledge in this demographic and to their reluctance to adopt new technologies. Rather than utilizing computer-based technologies and other touch screen devices, such as smartphones and tablets, research has shown that it can be more effective to utilize smart televisions as an assistive technology for older adults, to provide them with opportunities for social interactions. A study in Portugal has shown that integrating technology into already familiar objects can reduce this reluctance to adopt new supports [14]. Among all familiar objects, smart televisions appear to be one of the most promising. Smart televisions offer unique advantages that make them particularly suitable for enhancing accessibility and convenience in everyday life among older adults. Their widespread availability ensures that many older adults already have access to this technology, reducing the need for additional purchases or installations. The integration of smart televisions as assistive technologies into daily life has high potential, as many older adults already use televisions as a central part of their routine. This approach not only facilitates the adoption of new technology but also enriches the user experience without overwhelming them with unfamiliar devices. A study conducted by Wang et al [15] reported high television watching time among 252 older adults aged between 65 and 88 years, where approximately 25% of them spent more than 4 hours per day watching television. According to a study conducted by Alaoui and Lewkowicz [16], smart television applications to support socially oriented activities for older people have been designed. They propose that integrating smart television as an assistive device considers quality of life by improving their well-being and self-esteem [16].

### Objectives

To the best of our knowledge, despite the advantages offered by smart television design, its potential as an assistive technology for enhancing social communications among older adults has not been thoroughly explored. Thus, this study aimed to explore the state of the art in utilizing smart televisions as assistive technology for older adults to improve their communication and social lives.

## Methods

### Search Strategy

We performed the search based on the guidelines for performing a systematic literature review. A total of 6 databases were recommended: the IEEE, ACM, Google Scholar, ScienceDirect, Engineering Village by Brereton et al [17], and Springer by the Evidence-Based Software Engineering Technical Report [18]. This review was conducted without registering a protocol. The inclusion and exclusion criteria were defined before the search, as provided in Textbox 1.

**Inclusion and exclusion criteria for the study.**

**Inclusion criteria**

- Only articles published in the last 12 years were included; the concept of smart televisions is fairly new. Smart televisions only became mainstream in 2015. Before that, they were known as internet televisions [19], and there is a clear difference in what a smart television can do as compared with an internet television.
- The target group of research studies must be older adults aged ≥60 years; this is based on the World Health Organization definition of older adults [20].
- Articles must focus on how smart televisions are used as assistive technology, specifically as tools to improve the health, well-being, and quality of life of older adults. They must include an aspect of communication and social interaction among older adults.
- Articles must focus on assistive technologies that aim to provide a supportive living environment for older adults living at home or by themselves.
- Only peer-reviewed publications were included.
- Only English articles were included.

**Exclusion criteria**

- Articles where smart televisions are not used as assistive technologies, such as those purely focusing on entertainment without any implications on communication or social interaction, were excluded.
- Articles focusing on technologies, such as mobile phones, tablets, laptops, or any technologies other than smart televisions, were excluded.
- Dissertations, theses, and gray literature (eg, patents, research reports, and policy documents) were excluded.
- All non-English articles were excluded.

### Screening and Analysis

The search was conducted from February 20 to February 27, 2024. A range of keywords were used in different combinations, including “smart TV,” “older adults,” “elderly,” “communication,” “messaging,” “video call,” and “application.” The search yielded 6829 results: 169 (2.47%) from ScienceDirect, 13 (0.19%) from Engineering Village, 2 (0.03%) from the IEEE, 133 (1.95%) from the ACM, 4649 (68.08%) from Google Scholar, and 1863 (27.28%) from Springer. The screening process was conducted separately by the JL, CTC, and WKB. We removed 4158 (60.89%) duplicates and screened the remaining records. Their titles and abstracts were read for this round of screening, which resulted in the exclusion of 2610 (38.22%) records. The remaining 61 (0.89%) articles were read in full text to assess for eligibility. A total of 31 (51%) articles were excluded at this round of screening, and 30 (49%) articles were included. None of the articles were subjected to quantitative synthesis because of the significant variations in the data measured across the 30 included articles. Throughout the process, any disagreements were resolved through consensus.

On the basis of the aim of the systematic literature review, the included articles were analyzed according to the criteria described in Textbox 2.

**Criteria for analyzing the included studies.**

- Objective or objectives of the study
- Outcomes of the study, whether design, development, or framework or guidelines
- Methodology focusing on design
- Methodology focusing on evaluation
- New technology developed (what kind of technology and where it should be used)
- Impact on social interactions, whether direct or indirect
- Sample size and demographics (either during the design or evaluation process)
- User involvement; if yes, at which stage?
- Country or countries

## Results

### Study Selection

**Figure 1:**
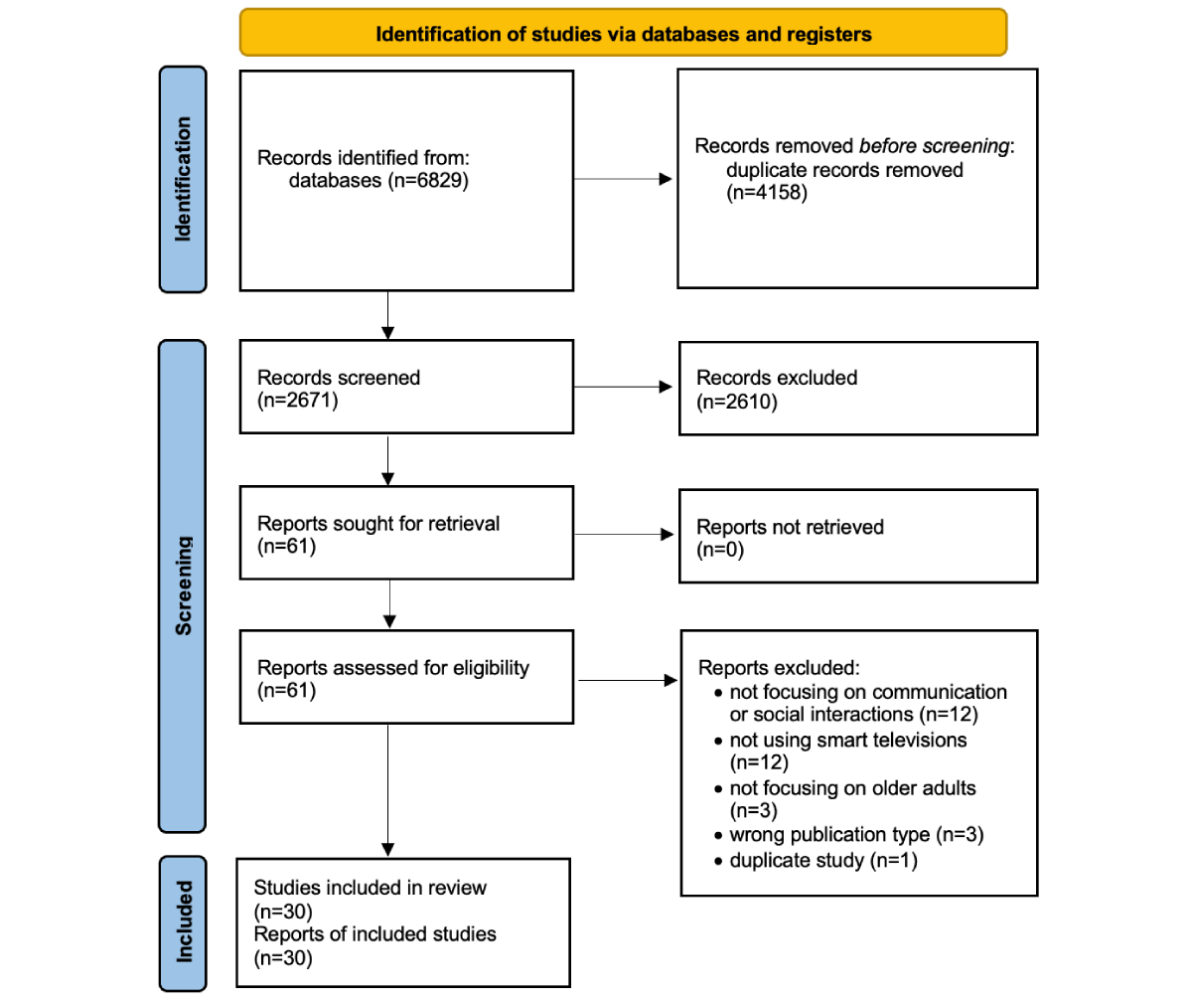
The PRISMA flow diagram.

Figure 1 depicts the entire process using a PRISMA (Preferred Reporting Items for Systematic Reviews and Meta-Analyses) flow diagram [29], providing details on the reasons for excluding certain articles along with their corresponding quantities. Table 1 summarizes the details of each of the 30 included articles according to the analysis criteria. Six studies are related to the fostering social interactions for better life experience (FoSIBLE) project, which aims to provide bridging spaces to foster social interactions and experiences among older adults [16,21-25]. Despite focusing on the same project, we included all 6 studies and identified them as relevant through the search and screening process, as the objectives and reported outcomes of these articles were not exactly the same. Other than the FoSIBLE project, López et al [26], López et al [27], Tapia et al [13], and Gutierrez et al [28] were also from the same project.

**Table 1:** Summary of the review results.

Study	Study objective	Methodology (design and evaluation)	Measured outcomes	New technology developed	User involvement and settings	Sample and country
Alaoui and Lewkowicz [16]	To investigate the use of interactive televisions for virtual networks and online generational communities as a means to compensate for the lack of relationships, prevent isolation, and enhance self-esteem among older individuals living alone at home.	Design: a living laboratory approach, including semistructured interviews and scenario presentation to end users, was conducted iteratively. Evaluation: proposed user testing using mock-up and focus group only; evaluation yet to be conducted.	The daily habits, lifestyle, and actual needs of participants in using television for group activities and communication, including product design, functionalities, and usability.	Yes, the FoSIBLE^a^ platform using HBBTV^b^. End users were able to use functions such as widgets, chat, book club, and vital date.	Yes, during the design phase; home setting.	A total of 10 older adults (8 females and 2 males), aged 65-90 years; France.
Alaoui et al [25]	To present the FoSIBLE and PaeLife^c^ projects, which propose solutions to support social interaction and social life among older adults. To describe the living laboratory approach and the ways in which the services were defined based on user analysis.	Design: a living laboratory approach, including semistructured interviews. Evaluation: usability testing for FoSIBLE and qualitative and observational analysis for PaeLife.	Accessibility of the application content and the device platform, feedback on the design of the device, and the interface of the smart television widget.	Yes, the FoSIBLE platform and the PaeLife application, a personal life assistant designed to enhance the social life of older adults.	Yes, during the design and evaluation phases; home setting.	The study included 10 older adults (8 females and 2 males), aged 65-90 years; France.
Alaoui et al [22]	To report the strategies and outcomes of implementing a UCD^d^ approach when designing interactive television-based services for older adults under the FoSIBLE project.	Design: (1) a UCD approach, including semistructured interviews and focus group, and (2) persona creation. Evaluation: iterative focus group sessions with mock-ups presented.	User feedback concerning needs, user acceptance, functionalities, and usability.	Yes, FoSIBLE platform using HBBTV.	Yes, during the design and evaluation phases; home setting.	The study included 10 older adults (8 females and 2 males), aged 65-90 years; France, Austria, and Germany (the authors noted that the interviews were conducted in all 3 countries, but the demographic information was only provided for the interviews in France).
Drobics et al [24]	To showcase the initial outcomes of the approach in using various multimodal interaction techniques for an integrated solution combining smart television with a social community platform, as part of the FoSIBLE platform.	Design: N/A^e^. Evaluation: End-user evaluation using a Kinect device.	Suitable mode of interaction and set of gestures.	Only specialized software components for gestures and a tablet interface to interact with the smart television were developed.	Yes, during the evaluation phase only.	The study included 24 participants, but no demographic details were reported; Austria.
Alaoui and Lewkowicz [21]	To contextualize the FoSIBLE project by highlighting the challenges of an aging population and the potential solutions offered by social television. To address issues related to the design and assessment of technologies through a living laboratory approach. To reflect on lessons learned and pinpoint recommendations.	Design: a living laboratory approach, including semistructured interviews. Evaluation: a 2-step evaluation; the first step was heuristic evaluation through expert testing sessions; the second was a system usability and usefulness assessment.	The usability of the proposed social television and the appropriateness of the design for older adults.	Yes, but no technical details were provided.	Yes, during the design and evaluation phases; home setting.	No information was provided about experts performing heuristic evaluations. The study included 5 older adults (2 males and 3 females), aged 67-92 years; France.
Müller et al [23]	To investigate the use of social television systems by older adults concerning how these systems interweave with sociocultural issues via a living laboratory approach and to reflect on the methodological approaches throughout the process.	Design: not reported in detail; previous user studies and a living laboratory approach were mentioned. Evaluation: a questionnaire, followed by individual demonstration sessions for usability testing; qualitative interviews were also conducted.	Questionnaire: an overview of the participants’ actual use of technologies. Demonstration sessions and interviews: the usability and functionality of the FoSIBLE program for older adults on social television.	Yes, Android television, with the FoSIBLE platform.	Yes, during the design and evaluation phases; home setting.	France: the study included 5 older adults (2 males and 3 females), aged 67-92 years. Germany: the study included 5 households comprising 9 individuals (all couples living together except for 1 single man; 6 males and 5 females).
Camargo et al [30]	To investigate whether linking intelligent personal assistants to television systems can enhance the social interactions of older adults with their family members, friends, and caregivers.	Design: not reported. Evaluation: a questionnaire and interviews.	The usability and acceptability of the proposed system, as well as perceptions of decreased feelings of loneliness among participants.	A prototype developed by integrating Google Assistant, Amazon Fire TV, and a notifications app for television.	Yes, during the evaluation phase only; home setting.	The study included 6 older adults, aged 64-90 years. Testing was conducted in Brazil; however, all authors were affiliated with a university in Portugal.
Coelho et al [31]	To propose the design of the SNSs^f^ that adopt technologies already familiar to older adults, such as televisions, to improve their ability to interact with these systems.	Design: (1) a population characterization phase using semistructured interviews and (2) a participatory design phase through focus groups. Evaluation: only user testing was proposed, but it had not yet been conducted.	Population characterization: user requirements to effectively use SNS technology, preference, and perspectives on using different technologies to access SNS. Participatory design: recommendations to improve the UI^g^.	You, me & TV prototype, providing 3 modalities (remote control, voice, and gesture) and 3 features (content publishing, content visualization, and group management) was created, but had not yet been fully developed.	Yes, during the design phase; home setting.	Population characterization: the study included 31 participants (20 female and 11 male), aged ≥60 years, approximately 93% of the participants had visual impairments, 36% had hearing impairments, and 33% expressed difficulty remembering past events. Participatory design: the study included 17 participants (14 female and 3 male), aged ≥60 years; Portugal.
Cortellessa et al [32]	To present ongoing work in designing and developing the TV-AssistDem solution, which aims to enable remote assistance for patients by using television-based data transmission and video interactivity among patients, family members, caregivers, and health care professionals.	Design: cocreation with potential end users through focus groups at different phases of the design and development process. Evaluation: (1) internal testing with health care professionals, experts, and caregivers for iterative improvement and (2) feasibility study with 2 subphases. The first phase involved usability testing (with data collection through observation, think-aloud protocols, and the SUS^h^ questionnaire). The second phase involved a prepilot study (including use observation and feedback collection).	Design: usability and functionality, specifically relating to existing and additional services for the management of dementia; improvements to the current UI; and identification of user needs (people with MCI^i^ and their formal and informal caregivers). Evaluation: (1) internal testing—iterative feedback regarding usability and functionalities aligned with user requirements and (2) feasibility study—assessment of the functionalities and usability of the TV-AssistDem platform, closely resembling the first phase of a larger-scale clinical trial, and assessment of the platform’s use in participants’ homes and its impact their well-being for the second phase.	Yes, the TV-AssistDem system which uses a set-top box, a webcam, and a dedicated remote control.	Yes, during the design and evaluation phases; home setting.	Design: (1) the study included 90 patients aged ≥60 years, who scored 23-27 points on the MMSE^j^ and had self-perceived or caregiver-perceived cognitive impairment that had been present for >6 months; and (2) health care workers specializing in dementia and cognitive decline, and informal caregivers. The number of participants who were health care workers and caregivers was not reported. Evaluation: (1) internal testing—the study included 7 participants comprising technology experts, usability experts, and health care professionals and (2) feasibility study—for the usability test, no details about the sample were reported other than the indication that it was a small group of representative users and a report on how they were involved. For the prepilot study, the study included 15 patients aged ≥60 years who scored 23-27 points on the MMSE and had self-perceived or caregiver-perceived cognitive impairment that had been present for >6 months. Design: Spain and Romania. Evaluation: Italy and Switzerland; the authors were from all 4 countries.
Costa et al [33]	To design, develop, and evaluate a smart television–based technology that delivers social and health services for older adults at home, while ensuring access to all services.	Design: requirements capture and analysis with stakeholders. Evaluation: a pilot test involving pre- and post-questionnaires with home users over a period of 7-15 days.	Design: identification of user requirements. Evaluation: usability, functionality, and acceptance. To validate the findings, the information gathered via questionnaires was compared with the data collected by the platform regarding the access of users to the system and applications.	Yes, hosted on a home theater PC (a computer designed to accompany a television set) and providing a variety of applications, such as social network clients, vital signs monitoring, interactive television contests, and conventional online care, including medication reminders and telemedicine.	Yes, during the design and evaluation phases; home setting.	Stakeholders: the social and health services of the regional government of Galicia, the most active senior associations, residential and day centers, in-home care solutions, and senior members of the engineering group responsible for the technical design. Older adults: 62 (50% male and female), around the region of Galicia. At least 65 years old (ie, retirement age in Spain at the time of the pilot). Spain.
Costa and Duarte [34]	To develop an adaptive multimodal fission component, integrated into the multimodal “Gentle UI for Elderly People” (GUIDE) framework, which aims to customize television-based application UI to suit specific user traits, enabling older adults and impaired users to interact through multiple output modes to address their interaction challenges.	Design: a UCD. User studies were conducted with end users during the early phases. Evaluation: user studies were conducted; however, no details were provided.	Usability, acceptance, and adoption of multimodalities when interacting with television-based applications.	Not precisely a technology but a framework, GUIDE was developed using hybrid television platforms and services, which include application platforms such as HBBTV and proprietary middleware solutions from television manufacturers.	Yes, during the design and evaluation phases; home setting.	Not specified; Portugal.
Doppler et al [35]	To develop “BRELOMATE,” a television- and tablet-based communication and entertainment platform that fosters user engagement and promotes social participation among older people, with a focus on adopting a participatory design approach.	Design: a UCD with iterations and a participatory design approach, including focus group and task-solving exercises. Evaluation: a field trial with time slots (4-6 participants in 6-12 weeks; because of overlap, there were once 19 participants simultaneously).	Robustness, applicability, and feasibility of the platform; usability of the platform and the online communication and entertainment services.	Yes, the “BRELOMATE” platform.	Yes, during the design and evaluation phases; home setting.	Design: the study included 4 females and 3 males; age was not specified. Evaluation: at the start, 33 participants were enrolled. A total of 3 participants withdrew because of installation problems. At the end, 15 participants (females and males), aged 50-84 years, completed the trial; Austria.
Dragić et al [36]	To present the “Home Health Smart TV” platform, which provides access to multimedia eHealth content customized for older adults and aims to address social isolation among them by offering video communications.	Design: not specified in detail; mentioned the involvement of both domains (eHealth and social networking) and described the platform’s features and physical elements. Evaluation: Not reported.	N/A, as the study focused solely on system design.	Not developed, but proposed applications for a smart television platform, including home, messages, education, measurement data, and social networking features on an Android television.	Not reported for user involvement; home setting.	N/A for the sample; Croatia.
Faria et al [14]	To understand how older adults perceive the use of the ProSeniorTV service, which aims to promote older adults’ participation in social activities held in their residential area and to help them remember to take their medication.	Design: not specified Evaluation: focus group sessions were held, where 2 specific scenarios were studied.	Participants’ perceptions of different scenarios when using ProSeniorTV, regarding the perceived usefulness of the program.	ProSeniorTV with use scenarios was proposed, but concrete development was not reported.	Yes, during the evaluation only; home setting.	The study included 6 Universidade Sénior de Cacia students (2 males and 4 females), aged 64-80 years (mean age 74 years; median 75 years). All were experienced ICT^k^ users; Portugal.
Gusev et al [37]	To propose the SMILE^l^ concept, which aims to develop a self-care social interactive television system as a service for older adults to support independent living and to prevent or delay dementia, by offering technologies, such as social networks, and personalized health monitoring.	Design: literature review and thematic analysis of relevant projects, focusing on usability and technological barriers.	N/A	No, only the SMILE concept was proposed, focusing on innovating models for service delivery by combining traditional IP television services with emerging technologies.	No user involvement; home setting.	N/A for the sample; Macedonia, the United Kingdom, and Slovenia.
Gutierrez et al [28]	To design and deploy the SocialConnector system, a computer-supported family communication mediator intended to identify key factors that ease the adoption of ambient intelligent systems, thereby promoting intergenerational family communication at home.	Design: a UCD with iterative and empirical experiments. Evaluation: task-based user testing, followed by participants completing the SUS questionnaire and participating in individual and focus group interviews. The same methods were used for the Social Connector hybrid version (smart television and tablet PCs), except that the SUS questionnaire was not applied.	Main barriers and perceived usefulness of the SocialConnector system.	Yes, the SocialConnector system, which can be used either on a smart television or in a hybrid format; allows older adults to connect with others using regular social media services.	Yes, during the design and evaluation phases; home setting.	The study included 8 older adult participants for both smart television and hybrid versions (4 females and 4 males). For the smart television version, participants were aged ≥60 and had low levels of technology appropriation. For the hybrid version, the participants’ ages were not specified, and they also had low levels of technology use and no prior interaction with tablet PCs and smart televisions; Chile.
Tapia et al [13]	To design and use a smart television application to promote social interaction among older adults and their family members through social media.	Design: not specified; only presented the design. Evaluation: heuristic inspection with expert users and usability study with end users (via think-aloud protocol and focus group discussion).	Heuristics inspection: usability issues identified before conducting user-based evaluation. Usability study: user experience and task completion success.	Yes, the SocialConnectorTV, consisting of an Android LED television equipped with a Google Chromecast device.	Yes, during the evaluation phase only; home setting.	Expert users: both authors and expert users; no further information was provided. End users: 8 older adults (4 males and 4 females), aged ≥60 years, with low-level technology appropriation; Chile.
Herrmanny et al, [38]	To design a system that supports the specific needs of older adults while preventing age-related isolation and loneliness by combining a social television community application with sensor-based and tangible social interaction.	Design: an extensive requirements analysis was conducted as part of a broader research project; no further details were provided. Evaluation: focus group sessions with hands-on interaction with the device and system, as well as group interview.	Technology acceptance, competence, and control among participants, as well as feedback on the design of the device and system.	Yes, a social television application developed for use with a Samsung smart television and a capacitive proximity sensor.	Yes, during the evaluation phase. The design phase is unknown, as no details were reported about the requirements analysis; home setting (senior housing facility).	The study included 15 older adults (10 females, 5 males), aged 62-77 years. Most participants watched television regularly; Germany.
Kakarountas [39]	To design a system that modifies familiar home appliances for older adults through commercial off-the-shelf products, so that HCI^m^ is hidden from them.	Design: a small survey. Evaluation: not conducted.	Not reported, as the article presented only technical descriptions of the proposed technology.	Yes, a smart television equipped with an Android installed HDMI^n^ dongle offering applications, such as weather information, video conferencing, and health monitoring.	Yes, during the design phase only; home setting.	The study included 4 males and 2 females, aged ≥70 years, diagnosed with various diseases, who expressed technology fear and were unwilling to learn new technologies; Greece.
Lee [40]	To identify the needs of diverse older adults and to determine which applications could improve their living conditions when introducing a smart home, with a particular focus on smart television.	Design: interviews combined with a questionnaire.	The perceptions of participants regarding the adoption of the smart home concept to retain family values and their demand for smart home services, including how manageable and adoptable digital house applications are, and how they manage related activities.	No, the study only explored the concept of a smart home integrated with a smart television, where users could share content with the family’s network and coordinate tasks with family partners such as supermarkets, schoolteachers, and health care centers.	Yes, regarding identifying needs.	No sample was reported; Taiwan.
Limdumrongnukoon et al [41]	To design and develop an interactive multimedia delivery system to distribute multimedia content among older adults, family members, relatives, friends, and health care providers.	A conceptual model was proposed, providing a system’s overview of interaction, functionalities, and technical specifications, based on existing literature and design guidelines.	N/A, as the study only involved a proposed conceptual model without validation.	No, the study only proposed a conceptual model.	Not reported for user involvement; home setting.	N/A; Thailand.
López et al [26]	To assess how patients with PD^o^ accept and use smart television and how health professionals and caregivers perceive this use. To evaluate the smart television games developed specifically for patients with PD, alongside their caregivers and health professionals.	Design and evaluation: testing was conducted individually and collectively, and focus groups were held with 3 user groups: professionals, caregivers, and patients.	Acceptance, usability, and engagement with the games.	Yes, cognitive games were developed and played on an Android television Box with minimum requirements: Android 5.0, HDMI, wireless remote control, and Ethernet or Wi-Fi connectivity.	Yes, during the design and evaluation phases; it was tested in care centers for patients with PD. The games were also suitable for those staying at home with caregiver assistance.	The study included 11 health professionals, 9 caregivers, and 16 patients with PD; Spain.
López et al [27]	To investigate the benefits of the proposed cognitive games on smart television platforms, including how continuous use affects cognitive abilities, how social aspects are influenced by collective gameplay, and how efficiently the cognitive status (ie, NCA^p^, MCI, and MD^q^) can be estimated.	Design: application of design guidelines and iterative testing with target users. Evaluation: an experimental setup in which participants played the cognitive games.	Acceptance, usability, and user experience in gameplay, including social factors such as comparing collective gameplay in care centers with individual gameplay at home. Clinical effect was assessed through cognitive exercise and socialization outcomes.	Yes, cognitive games were developed as smart television applications, along with an automatic screening tool and a remote control. These applications were installed on an Android box connected to a television via HDMI.	Yes, during the design and evaluation phases; hospitals, day care centers, and patients’ homes.	France: the study included patients with early-stage AD^r^ or similar dementia and their caregivers. Spain: the study included patients with PD, caregivers, health professionals, social workers, and administrators. Hungary: the study included patients with AD, caregivers, health professionals, and doctors. The participants were divided into 3 cognitive ability groups: MD —with 14 participants, 5 patients (2 males and 3 females), based on the MoCA^s^ scales, aged 71-80 years; MCI—with 26 participants, 12 patients (5 males and 7 females) based on the MoCA, aged 62-80 years; and NCA—with 45 participants, 5 patients (2 males and 3 females) based on the MoCA, aged 62-80 years.
Macis et al [42]	To propose a framework for a novel ICT system coupled with smart television to support active aging. In doing so, the authors outlined the rationale for developing the proposed framework and presented the current state of the system, along with possibilities for future expansions that are natively supported by the framework.	Design only: Desk and field research; user sessions conducted in 3 European countries using a user profiling questionnaire and a usability study (observation, think-aloud protocols, and post-session questionnaires). Older participants were invited and encouraged to work and engage with a variety of existing service platforms.	Demographic background that is linked to the usability of different systems, with potential for using those systems, including the willingness to adopt such service platform, the perceived added values, and the price participants were willing to pay.	A proposed prototype using an Android television box and a television with HDMI connectivity, but the system was not fully developed.	Yes, during the design phase; home setting.	The Netherlands: the study included 13 older adults (average age 71.24, SD 6.88 years; 8 male and 5 female; relatively highly educated). Belgium: the study included 13 participants (average age 78.15, SD 4.26 years; 6 males and 7 females; relatively highly educated; less ICT use than Dutch participants). Italy: the study included 29 participants (14 males and 15 females; average age 70.1, SD 5.8 years; less ICT use than Belgian participants)
Ribeiro et al [43]	To aggregate technological solutions supporting home care by exploring the use of a television set as the central device for interaction and communication at home. More precisely, the article aimed to investigate how the proposed solution, Active Assisted Living (AAL@MEO), could be advantageous for older adult who are not familiar with technological advances but are used to controlling their television set.	Design: not reported. Evaluation: 3 stages— pretest, during-test, and posttest. The pretest involved the application of a demographic questionnaire to characterize participants. During the test, the participants completed several tasks using AAL@MEO. The posttest involved completing usability and evaluation instruments.	Usability; the primary focus was on critical incidents observed when participants completed tasks and how they performed these tasks. The incidents were categorized as authentication, interaction using the remote control, menu navigation, use of measurement instruments, measurement confirmation, general application behavior, and user-application interaction.	Not specified, but participants tested the AAL*MEO prototype.	Yes, during the evaluation phase; the settings were not specified, but the collaborators involved in the study were those providing care to older adults.	The study included 30 collaborators (average age 58 years; oldest participant aged 67 years and youngest aged 54 years). All the participants were female, with varied education levels, although the majority had completed the fourth grade; Portugal.
Wang [44]	To assess the usability and functionalities of a virtual fitness platform on smart television with multiple users.	Design: design guidelines were presented. Evaluation: Two experiments—(1) usability testing with a questionnaire and (2) longitudinal field study with observation and interview.	The system functionality and usability, including use, interaction with others, motivation, perceived ease of use, and perceived usefulness.	Yes, a fitness platform that enables multiuser virtual situations on a smart television using an Android television system.	Yes, during the evaluation phase; home and care centers.	The study included 40 older adults aged 60-80 years (6 at home and 34 in 3 day care facilities); Taiwan.
Wang and Wu [45]	To explore the digital experience of older adults concerning digital feedback, technology anxiety, and the influence of existing familiar skills on their digital habits. More precisely, to use digital devices (ie, smart television) to overcome the digital divide and demonstrate how such use may impact intergenerational relationships.	Design and evaluation: N/A; a questionnaire was used for data collection. Data analysis was conducted using the partial least squares method for structural equation analysis.	Demographic information; associations between technology anxiety, digital feedback, familiarity with technology, technology habits, and intergenerational relationships.	No.	N/A	Older adults aged >55 years were invited to complete the questionnaire. The study included 233 male and 246 female participants. Approximately 50% of the participants were aged 60-69 years, 20% were aged 55-59 years, and the remainder were aged ≥70 years. A total of 43% of the participants had completed elementary school, 24% had completed junior high school, 20% had completed senior high school, and the remainder had completed junior college or higher. Forty percent were retired; Taiwan.
Wu and Hu [46]	To examine the possibilities for older adults in using intelligent television. To investigate significant differences in physiological and psychological characteristics among older adults living in small cities and big cities. To identify existing issues and the real needs and preferences of older adults when using intelligent television; and to provide important references and theoretical grounds for markets and relevant stakeholders in this area.	Design: the study involved the application of the 5W2H (Who, What, When, Where, Why, How, and How Much) design and analysis, and participatory design through questionnaires and in-depth surveys with participants from different cities. Evaluation: N/A; the study involved only investigating possibilities.	Using the developed “3E” (easy and efficient for the elderly) model, the researchers aimed to measure usability (ie, whether intelligent television was easy and efficient for older adults) and satisfaction.	N/A; the study only constructed a theoretical framework, that is, the “3E” model, based on the iterative innovation design method and outcomes.	Yes, during the design stage; home setting.	Questionnaires: a total of 150 questionnaires were distributed in 3 groups of older adults aged >60 years living in new urban areas, small cities, and big cities; only 117 valid responses were collected. In-depth surveys: 3 participants from Maanshan (a typical case of urbanization development), 1 from Wuhua (a small city with a smaller population), and 1 from Guangzhou (a big city); China.
Naudé et al [47]	To identify the barriers and enablers for older adults in using DiTV^t^, which could help identify potential action levers to improve the implementation of the DiTV system and its telecommunication functionalities.	Design: not reported; the DiTV system, e-lioTV, had already been developed before the study. Evaluation: case study with observations of participants performing tasks (older adults only) and semistructured interviews.	The stages of DiTV integration in geriatric institutions; the role of care professionals in this; and the perspectives and user experiences with the DiTV system.	The DiTV system, e-lioTV, had already been developed by a start-up company; further details were not provided in this study.	Yes, during the evaluation phase; geriatric care.	The study included 18 residents (11 females and 7 males) aged 68-90 years. Six care professionals (3 sociocultural animators, 1 deputy director, and 2 technical managers) living or working in nursing homes also participated; France and Ireland.
Santana-Mancilla and Anido-Rifón [48]	To examine the acceptability of interactive television care by older adults and its suitability and potential for improving their quality of life in an age-friendly environment.	Design: UCD. Evaluation: the study used interview, user testing, and a questionnaire incorporating the technology acceptance model.	Technology acceptance, system usability, and user attitude.	Yes, an interactive television care platform using Google TV.	Yes, during both the design and evaluation phases; home and nursing home settings.	The study included 50 older adults during the pilot test. A total of 500 older adults and caregivers participated in interviews, but no details were provided regarding the process and their involvement; Mexico.

^a^FoSIBLE: fostering social interactions for better life experience.

^b^HBBTV: hybrid broadcast broadband television.

^c^PaeLife: personal life assistant.

^d^UCD: user-centered design.

^e^N/A: not applicable.

^f^SNSs: social networking systems.

^g^UI: user interface.

^h^SUS: system usability scale.

^i^MCI: mild cognitive impairment.

^j^MMSE: Mini-Mental State Examination.

^k^ICT: information and communication technology.

^l^SMILE: stimulating intellectual activity with adaptive environment.

^m^HCI: human-computer interaction.

^n^HDMI: high-definition multimedia interface.

^o^PD: Parkinson disease.

^p^NCA: normal cognitive abilities.

^q^MD: mild dementia.

^r^AD: Alzheimer disease.

^s^MoCA: Montreal Cognitive Assessment.

^t^DiTV: digital interactive television.

### Study Characteristics

More than half of the studies explicitly focused on the context of home care. The studies that were not included in this category either did not specify a context or were conducted in geriatric care units or nursing homes. For instance, Naudé et al [47] focused on identifying enablers and barriers to the use of digital interactive television (DiTV) in nursing homes. According to Naudé et al [47], DiTV is a system that allows viewers to engage actively with content in real time. Examples of this include online shopping via television and being able to vote or comment on social posts via television, with the latter being considered a form of asynchronous communication.

More than half of the studies focused on designing or evaluating attitudes toward technology and assistive technologies, while the remainder focused on proposing concepts and frameworks. Wu and Hu [46] discussed the 3E model, known as easy and efficient for the elderly. This model was developed to better study behaviors related to the use of smart televisions among older adults. The discussion of this model was presented in a theoretical manner to analyze its framework. Although not focused on the 3E model, Gusev et al [37] proposed the stimulating intellectual activity with adaptive environment (SMILE) framework, which aims to prolong independence and improve the quality of life through cognitive stimulation, social inclusion, and personalized self-care systems. Various projects focusing on support for older adults were analyzed, including medical sensors installed in home products; the use of mobile phones and smartphones as personalized devices; and the Vital Mind project, which aims to increase the lifespan of older adults by enabling them to participate in cognitive fitness exercises actively while watching television.

Regarding the sample size of the participants, 5 articles did not report any sample size or demographics [34,36,37,40,41]. The sample sizes ranged from studies with only 5 participants to studies with more than 200 participants per sex. These variations were related to the differing methodologies of the articles. The studies that focused on assessing the accessibility of content and device platforms typically included a small number of participants, as demonstrated in the study by Alaoui et al [25], whereas studies that focused on measuring technology habits, technology skills [42], or intergenerational relationships [45] included larger sample sizes. Results from larger sample sizes were obtained through surveys as the mode of data collection. For example, Wang [44] and Cortellessa et al [32], who used surveys to measure attitudes, included between 30 and 100 participants. Geographically, all but 8 studies were conducted in Europe. Other studies were conducted in China [46], Thailand [41], Taiwan [40,44,45], Chile [13,28], and Mexico [48]. No studies from other regions, such as Africa and North America, were included.

Similarly, not all articles provided information about the participants. Most articles specified the age, gender, and demographics of their study population. The participants across all included studies ranged in age from 50 to 92 years, but most information was drawn from the participants aged 60 to 80 years. Some studies were conducted in the context of geriatric care units or nursing homes [26,27,38,44,47,48]. In these facilities, care professionals and older adults living there were interviewed regarding the DiTV system that was implemented [47]. In addition to Naudé et al [47], López et al [26] and López et al [27] also specifically mentioned the inclusion of administrative staff, ranging from health professionals to formal and informal caregivers.

The topics included in the articles covered different aspects of utilizing smart televisions as assistive technology for older adults. Most of the articles reported some form of newly developed technology, while 6 articles reported no technology development [30,37,40,43,45,47]. The technologies that were proposed and developed across all articles were various forms of interactive television. These included Android televisions and Google televisions; almost half of the included studies did not specify the type of smart television proposed or developed.

Approximately half of the articles were directly related to the social aspects of health in older age. The other half was indirectly related, addressing social health through other factors. For example, Wang [44] examined the usability of a virtual fitness platform on smart televisions for health promotion among older adults. This was indirectly related to social health because older adults were given the opportunity to interact with others via the virtual fitness platform, even though its purpose was to improve physical health.

## Discussion

### Principal Findings

To the best of our knowledge, this review is the first to summarize the state-of-the-art research on the use of smart televisions as assistive technologies for communications and social life among older adults. Andreadis and Zambon [49] in their survey, provided an overview of compelling studies that used television devices as the primary user interface to address the social exclusion and digital barriers encountered by older adults. However, almost all studies included in their review involved the use of traditional televisions, rather than smart televisions. In many other studies, smart televisions have been used to collect data to assist with everyday life activities (eg, reminders, health status monitoring, and calendar management) [50,51]; as smart decision support systems to support healthy aging [52]; and as rehabilitation tools [53]. Although some of these studies incorporated certain social elements, such as shared training and gaming activities, their primary focus was not on fostering social communications among older adults to reduce exclusion and loneliness. Wong et al [54] published a scoping review protocol to provide an overview of existing research findings on factors that aid or hinder the use of smart televisions by older adults in care settings. However, at the time of writing this review in November 2024, their scoping review remained unpublished. Another review conducted by Carvalho et al [55] reported solely on television remote controls and their use by older adults. Their findings included usability issues faced by this user group when using television remote controls and proposed solutions to address these issues.

### Comparison With Prior Work

#### Evidence and the Number of Included Studies

Although 30 relevant studies were included in this review, this number indicates that smart televisions have been seldom utilized as assistive technologies to enhance communication and social lives among older adults. Despite several of the included publications stemming from the same or larger studies, it is notable that these publications involved participants from multiple countries. Conversely, some publications that were not part of larger studies also included participants from different countries. For example, Alaoui et al [22] conducted interviews with participants living in France, Austria, and Germany, while Müller et al [23] included participants from both France and Germany. In addition, Gusev et al [37] involved users from Macedonia, the United Kingdom, and Slovenia when proposing a socially interactive television–based self-care system tailored for older adults to support independent living and aid in the prevention or delay of dementia. These studies show that the same technology developed has potential for use across diverse geographical areas.

When considering the countries involved in the included studies, the majority were conducted in European countries or other technologically advanced countries. This geographic concentration may limit the generalizability of the findings, particularly in non-Western or resource-limited settings where cultural, economic, and technological factors differ significantly. Our findings indicate that no studies from Africa or North America were represented. This is unsurprising, as research on more advanced technologies has historically been concentrated in regions with greater access to resources, infrastructure, and technological expertise. This aligns with the work of Ondiek and Onyango [56] and Bosch and Currin [57], who highlight both the challenges and efforts involved in engaging user groups from these underrepresented regions. Therefore, when generalizing these findings, it is crucial to consider the unique challenges and needs of such regions, as the effectiveness and applicability of the technology may vary.

As most studies focused on designing, developing, and evaluating prototypes, many also proposed some design guidelines [27,44]. These guidelines may be useful not only for researchers and policy makers, but also for designers working in this field. Almost all articles reporting on prototypes indicated that the technology had been developed by the researchers themselves. By contrast, Carvalho et al [55] was one of the few studies to include a smart television application, e-lioTV [58], which was developed by a private start-up named Technosens [59]. Costa et al [33] also involved an engineering group responsible for technical design in their study. These findings indicate that there may be a lack of collaboration between the public and private sectors, which could potentially limit the use of smart televisions as assistive technologies for enhancing social interaction among older adults.

Finally, it is worth mentioning that some of the included articles did not prioritize social communication as the main aspect of their functionalities. However, they highlighted the impacts such functionalities had on influencing the social lives of older adults. For instance, Wang [44] proposed a virtual fitness platform on smart television with multiple users, and Gusev et al [37] and Lee [40] proposed self-care interactive smart television applications that incorporated social interactions as one of their features. We acknowledge that social interactions can occur in various forms—both synchronous and asynchronous—and with different people, including peers, family members, and formal and informal caregivers. For this reason, these articles were included in this review.

#### User Involvement

Older adults often face unique challenges related to mobility, sensory perception, and cognitive function, which can vary significantly from person to person [60,61]. Involving older adults in the design of assistive technologies is essential for creating effective and user-friendly solutions tailored to their specific needs. By including them in the design process, developers can gain valuable insights into their preferences, limitations, and everyday experiences, ensuring that these technologies are relevant and accessible. Research has shown that user involvement of older adults has been low in the design of assistive technologies intended for this user group [62]. However, our findings indicate that user involvement has been high regarding the utilization of smart televisions as communication-type assistive technologies for this group. Among the 30 included studies, almost all involved users during the design and/or evaluation phases. A few articles specifically mentioned that they adopted a user-centered design approach [22,28,34,35,48]. From these studies, it was evident that feedback from older users contributed to identifying potential usability issues and informing design features that truly accommodate their capabilities and needs. It is worth noting that involving older adults can promote a sense of ownership [63] and acceptance of the proposed technology [62]. This may also contribute to the scalability of the use of technology [64].

These articles not only demonstrate user involvement, but also show careful considerations when involving older adults. One of the reasons for utilizing smart televisions as social communication–type assistive technologies is because of their familiarity. Hence, older adults with low technology use have been one of the main target groups. Gutierrez et al [28] and Tapia et al [13], in their studies, specifically targeted older adults with low technology use. In addition to this primary group of end users, some studies also involved secondary end users. Cortellessa et al [32] conducted initial internal testing to gather feedback from experts, caregivers, and health care professionals. This internal testing aimed to inform iterative technological refinements before engaging end users, which could enhance the prototype’s technical stability, usability, and design quality before involving older adults and proceeding to a feasibility study. When the proposed technologies were intended for use by older adults with dementia, health care personnel and caregivers (formal or informal) were involved as well [27,32].

#### Attitudes in Using Smart TVs for Communications

Older adults often hold diverse attitudes toward using smart televisions as a means of communication, shaped by factors, such as familiarity with technology, perceived usefulness, and social needs. Depending on their backgrounds, some older adults have more proficient digital literacy than others. Age has been shown to be one of the strongest predictors in online participation, but race, gender, and education play a role as well [65]. In Finland, 80% of people aged between 65 and 74 years have used the internet, and 57% use it daily [66]. However, these numbers drop to approximately 41% of internet use and 23% of daily use among adults aged between 75 and 89 years. This difference in internet use highlights discrepancies in digital literacy and varying attitudes toward technology based on age. In addition, a study found that females were more likely to show lower global and technical confidence and to have lower operational and less positive attitudes toward technology compared with males [67]. Generally, older adults tend to feel more comfortable operating a television with a remote control than a smartphone or tablet. Therefore, we believe that many older adults will hold more positive attitudes toward a smart television interface as a means of improving social connection.

### Limitations

Although this study contributes to the understanding of the state of the art in using smart televisions to improve social connectivity among older adults, several limitations must be acknowledged when interpreting the findings. First, the study only included literature published in English. By only reviewing articles written and published in English, important findings from studies conducted in other languages may have been missed. Second, articles published more than 10 to 12 years ago were excluded. As mentioned in our inclusion and exclusion criteria, the concept of smart televisions is relatively new. While this ensures that the review focuses on recent advancements, it may also limit the inclusion of foundational research that could provide context for the transition of smart televisions into assistive technologies. Third, only peer-reviewed literature was included. The exclusion of gray literature may result in missing valuable information that is not traditionally found in academic publications. While this study focused on peer-reviewed literature, future studies could incorporate gray literature, such as patents, white papers, ongoing research, and dissertations, to gain a broader understanding of the real-world applications of smart televisions as an assistive technology [68].

The next limitation of this study is the potential exclusion of relevant studies because of the search strategy. Although we carefully selected search terms to capture both the direct and indirect impacts of smart televisions on the social lives of older adults, there may still be relevant articles that used different terminology. Consequently, some studies that explored the social implications of smart televisions in alternative ways may not have been included. Expanding the search strategy in future reviews by incorporating a broader range of keywords and synonyms could help address this limitation and provide a more comprehensive understanding of the topic. Regarding the generalizability of the findings, it is important to note that the geographic concentration may have affected the results, particularly concerning the non-Western or resource-limited settings. As mentioned earlier, most of the included articles were from Europe, and European contexts often differ culturally, economically, and technologically from other regions. Therefore, the applicability of the findings to diverse global populations may be limited. Finally, this review was conducted without registering the protocol. While this practice is not mandatory, we acknowledge this as a limitation.

### Conclusions

With people in today’s society living longer, the shift toward an aging population is undeniable. As people age, significant declines in both physical and cognitive health are common and are often accompanied by an increased risk of social isolation, which can further impact well-being. Because depression or the onset of depressive symptoms is a prevalent issue among older adults, introducing assistive technologies to enhance their communications and social life is crucial for supporting holistic well-being. While numerous assistive technologies present usability challenges for older adults, smart televisions stand out as one of the most familiar and frequently used technologies within this demographic. Thus, this research is essential for understanding the potential of smart televisions as an assistive technology to enhance social connections and communication among older adults, thereby addressing the challenges of exclusion and loneliness.

This review reveals that smart televisions hold significant promise as an assistive technology to enhance social connections and communication for older adults. The number of articles included in this study is 30, which is relatively low considering that smart televisions have been on the market for more than 10 years. Of these 30 studies, 6 were associated with the same project, that is, the FoSIBLE project [16,21-25]. In addition, López et al [26], López et al [27], Tapia et al [13], and Gutierrez et al [28] were from the same respective studies. Many studies examined the use of smart televisions for social connectivity in home settings; however, some also explored their application in senior housing facilities, care centers, hospitals, and nursing homes, highlighting their potential across diverse environments.

Most of the articles focused on user attitudes toward the design and evaluation of the technology, while others focused on conceptual and framework proposals. Of the 30 articles, 24 either proposed or developed interactive smart television applications and platforms that used a variety of smart televisions, including Google and Android. A wide range in participant sample sizes was observed, varying from 5 to more than 200 participants, depending on the research methodology. In addition, not all articles provided detailed information about the participants. User involvement emerged as a key factor in the development and evaluation of the proposed technologies. Although research has traditionally shown low user involvement in designing assistive technologies for older adults, this review indicates that most studies integrated older users at various stages of the design and evaluation. Finally, the review highlights the distinction between the direct and indirect contributions of smart televisions to the social lives of older adults.

While this review provides valuable insight into the role of smart televisions as assistive technologies for enhancing the social connectivity of older adults, several research gaps remain. First, most of the studies focused on short-term usability and initial user experience. There is little research on how smart televisions can impact and change the trajectory of the social well-being of older adults over time. Future studies should conduct longitudinal research to assess the long-term impact of smart televisions on the social well-being of older adults. This includes examining whether smart televisions can be used sustainably over extended periods and whether their sustained use can foster social engagement and improve quality of life over time. Second, 22 studies were conducted in Europe, 5 in Asia [40,41,44-46], and 3 in South America [13,28,48], with no representation from North America and Africa. This underscores the need for future research to engage participants from underrepresented regions to ensure that findings are more globally applicable and inclusive of varying needs, preferences, and challenges. To address this knowledge gap, cross-cultural research should also be conducted. Future studies should focus on these underrepresented regions to understand how different cultural attitudes toward technology and access to smart televisions may influence the adoption of assistive technologies for older adults.

Third, while some studies have explored how smart televisions can be used as assistive technologies for social connection among older adults with specific health conditions, such as Parkinson disease, dementia, and other health-related challenges, research on adaptation for these users remains limited. There is limited information on long-term adaptation for improving their social connectivity. Future research should explore adaptive accessibility solutions and features in smart televisions that evolve with users’ changing abilities and assess the long-term impacts of smart televisions on social engagement and quality of life for older adults with diverse needs. These solutions and features may include voice commands, improved remote control design, and other innovative interaction methods and modalities for interacting with smart televisions. By prioritizing these solutions and features, researchers may better address the diverse health conditions of older adults. In addition, bridging these research gaps to reduce barriers to adoption is essential for developing a more comprehensive understanding of how smart televisions can support long-term social engagement, be adopted across diverse cultural contexts, assess their actual impact on the social well-being of older adults, and adapt to the progressive needs of older adults with health conditions. In addition, future research should explore how smart televisions could serve as more accessible assistive technologies for older adults with disabilities. Smart televisions may be easier to interact with compared with smaller touchscreen devices. For instance, a remote control with larger buttons may be more manageable for older adults with disabilities than small touch-sensitive devices such as smartphones and tablets.

We hope that our review will inspire others and pave the way for future research exploring the potential of smart televisions as assistive technologies. These technologies hold promise for fostering meaningful social interactions, reducing social isolation, and enhancing the health, well-being, and overall quality of life of the aging population. Stakeholders, such as policy makers, decision makers, and designers, can also refer to our work, as it provides a comprehensive summary of the state of the art in this field. By understanding the current advancements and gaps highlighted in our review, they can develop strategies and design solutions that are better aligned with the needs and preferences of older adults.

## Appendix

Multimedia Appendix 1 PRISMA checklist.

## Abbreviations

DiTV: digital interactive television
FoSIBLE: fostering social interactions for better life experience
PRISMA: Preferred Reporting Items for Systematic Reviews and Meta-Analyses
SMILE: stimulating intellectual activity with adaptive environment

## Data Availability

The data and other materials used in this study are available upon request from the corresponding author, WKB.
